# Middle-Ear Microsurgery Simulation to Improve New Robotic Procedures

**DOI:** 10.1155/2014/891742

**Published:** 2014-07-23

**Authors:** Guillaume Kazmitcheff, Yann Nguyen, Mathieu Miroir, Fabien Péan, Evelyne Ferrary, Stéphane Cotin, Olivier Sterkers, Christian Duriez

**Affiliations:** ^1^INSERM, “Minimally Invasive Robot-Based Hearing Rehabilitation”, UMR_S 1159, 75005 Paris, France; ^2^Sorbonne University, UPMC Univ Paris 06, UMR_S 1159, 75005 Paris, France; ^3^Shacra, INRIA, University Lille 1, 59650 Villeneuve d'Ascq, France; ^4^AP-HP, Otolaryngology Department, Unit of Otology, Auditory Implants and Skull Base Surgery, Hospital Pitié Salpêtrière, 75013 Paris, France

## Abstract

Otological microsurgery is delicate and requires high dexterity in bad ergonomic conditions. To assist surgeons in these indications, a teleoperated system, called RobOtol, is developed. This robot enhances gesture accuracy and handiness and allows exploration of new procedures for middle ear surgery. To plan new procedures that exploit the capacities given by the robot, a surgical simulator is developed. The simulation reproduces with high fidelity the behavior of the anatomical structures and can also be used as a training tool for an easier control of the robot for surgeons. In the paper, we introduce the middle ear surgical simulation and then we perform virtually two challenging procedures with the robot. We show how interactive simulation can assist in analyzing the benefits of robotics in the case of complex manipulations or ergonomics studies and allow the development of innovative surgical procedures. New robot-based microsurgical procedures are investigated. The improvement offered by RobOtol is also evaluated and discussed.

## 1. Introduction

Surgical robot-based systems raise a great expectation for medical care. These systems are designed to improve quality and safety of surgical interventions and to lead to clinical benefits for the patient such as a reduction of the hospitalization duration [1]. Moreover robot-based and computer assisted surgeries enhance conventional gestures, with improved ergonomics and accuracy. In some case, as discussed in this paper, they even allow innovative procedures. However, when the use of robot changes the clinical practice, new procedures need to be designed, evaluated, and taught before clinical application. The aim of this work is to show that it is possible to use the simulation to design, train, and develop new robotic procedures.

**Figure 1:**
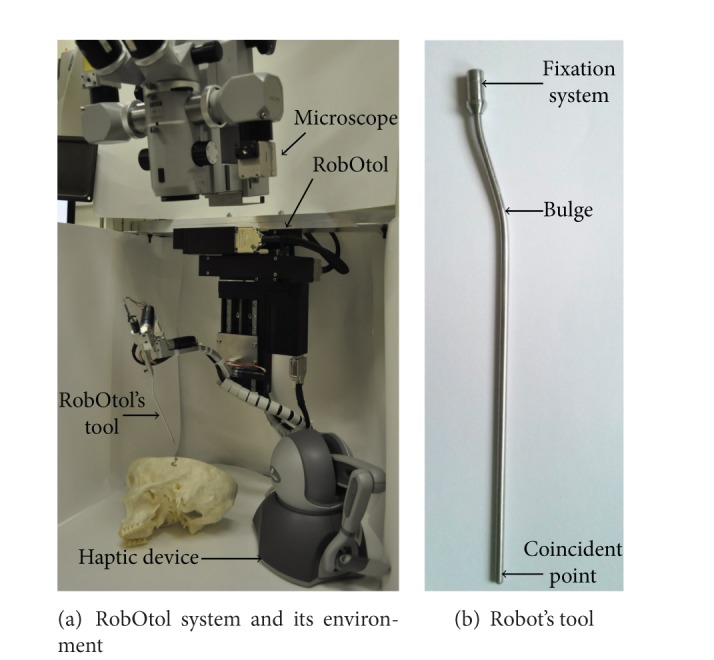
RobOtol, a teleoperated system for middle ear microsurgery. (a) The surgeon uses a microscope and commands the robot with the haptic device, a Phantom Omni (Sensable, Wilmington, MA). (b) To avoid visual obstruction by the robot, a bulge is designed on the tool.

In this work, we have focused on middle ear microsurgery. Microsurgery is an excellent scope for robotic systems [2–4] and several robots have been designed for middle ear microsurgery [5, 6]. This work is based on a teleoperated system called RobOtol [7] (Figure 1(a)). The RobOtol system is developed in order to avoid reduction of the surgical field exposure by the surgeon's hands, to stop physiological tremor [8], and to raise gesture accuracy.

**Figure 2:**
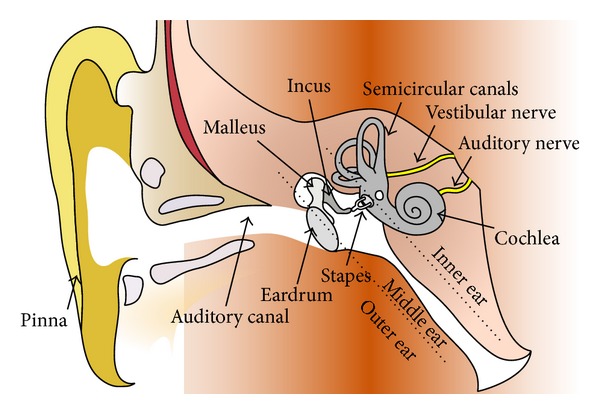
Human ear in surgical condition. Surgeons use a speculum to enlarge cartilaginous part of the external ear canal and may use up to 2 tools.

The middle ear ensures the mechanical transmission of the sound wave from the tympanic membrane to the stapes footplate through the ossicular chain which conducts the sound wave to the inner ear. It is located between the outer and the inner ear and is composed of the tympanic membrane, the ossicles (malleus, incus, and stapes), the middle ear cleft, and mastoid cells (Figure 2).

When a patient suffers from otosclerosis, the stapes footplate is progressively fixed to the inner ear, which leads to conductive hearing loss. The treatment such as ossiculoplasty consists in the replacement of one or several ossicles by prosthesis during a microsurgery. When performing the conventional surgery through the auditory canal, surgeons have to manipulate bones of less than 4 mm high localized inside a maximized 16 × 16 mm cavity size at 34 mm depth. Yet, several structures including the ossicles and the facial nerve are very sensitive to injury. To perform a nontraumatic intervention, submillimetric motion inside a narrow workspace is required. Consequently, conventional gesture remains complicated and stressful for surgeons.

The use of the RobOtol system should reduce the risks of the surgery and increase physicians' confidence. The benefits of the robot were evaluated by 4 surgeons (seniors and juniors) in [9, 10]. The robot is currently being evaluated to get the approval to carry out the first clinical trials. In this context, we propose developing a new tool, based on simulation, which has two objectives: the first is to provide a simulator to adapt the robot-based procedure before a real clinical use. Consequently, the procedure can be rehearsed to fully benefit from the use of the robot and the final design of the tools of the robot can be adjusted. The second is to develop a training tool for physicians to get familiar with RobOtol handling. Since new surgical procedures and gestures are involved, it is important for surgeons to master the robot arm. Thereby virtual simulations of those complex systems are developed for practice essentially. Nevertheless, real-time simulation is mandatory to interact realistically with a system and to obtain direct outcomes from the simulation.

The use of surgical simulators is often targeted towards training of beginners. Using virtual reality technologies, the environment of the procedure is numerically reproduced. The trainee can interact with the virtual anatomy and even get force-feedback using haptic technologies. For instance, the Visible Ear [11] and the Voxel-Man TempoSurg (Voxel-Man Group, Hamburg, Germany) are currently available to simulate only the mastoidectomy surgery. This procedure consists in drilling of temporal bone and does not involve interaction with the ossicular chain. Several finite element models of the human middle ear are reported in the literature. Their goals are to analyze and reproduce the behavior of the ossicular chain with different configuration, such as intact or pathological cases [12, 13]. Those studies are not real-time simulation and thus are not suitable for training.

The main contribution of this paper is to use the surgical interactive simulation to improve the robot-based procedures: we propose using this simulator to adapt the surgical procedure and to improve the design of the robot and its tools. Moreover, the simulation is used as a training tool before clinical translation. Thus, training is not only for beginners but also for the expert surgeons that will perform the first clinical trials. The key idea is that they need to get accustomed to the robot commands, before the first use in clinic. To do so, the simulation needs a precise modeling of the middle ear structure behavior. It is based on a finite element model (FEM). The mechanical interactions with instruments of the robots (and especially the contact response) are treated using constraint-based approaches. Moreover, the simulation has been optimized in order to reach real-time computations during the simulation. The drilling of the stapes footplate and the placing of a snap-in ossicular prosthesis on the ossicles constitute two challenging surgical procedures of the middle ear to perform in reality or to simulate.

We describe in this paper the implementation of the surgical simulation to model these two procedures with the robot. We show that the simulation is able to reproduce faithfully both of them and can improve and evaluate the robot-based procedures.

## 2. Material and Methods

### 2.1. RobOtol Description

RobOtol is a teleoperated system with 6 degrees of freedom, which is controlled by a Phantom Omni device (Sensable, Wilmington, MA) as a master arm (Figure 1(a)). Robot actuation is performed by a *XYZ* cross table and 3 rotary actuators with a coincident intersection point localized at the tip of the robot's tool (Figure 1(b)). An operative microscope with a focal distance of 300 mm is placed above the robot with its axis of view collinear to the *Z* linear stage and to the external ear meatus.

The design and the kinematics of the robot were optimized in order to reach all of the area of the middle ear, to maximize the operating field of view and the distance between the robot's body and the patient (for safety reasons) [7, 9]. The haptic device is only used as position input system since no force sensor is present on the robot.

A dead man footswitch is used to enable the actuators and to confirm the command. The command of the robot is described in [10] and is based on the equivalence relation of the master arm stylus and the robot's tool. The position-position command was used for all the experiments in this study. Thus, the robot moves to follow the master arm stylus position and stops when it reaches the target or when the surgeon releases the dead man footswitch. A homothetic parameter set at 7 for the translation and 1 for the rotation was implemented between the master and the slave arm for ergonomic reason and to gain in accuracy. The robot is able to perform delicate tasks with high dexterity in a narrow workspace.

### 2.2. Interactive Simulation Description

**Figure 3:**
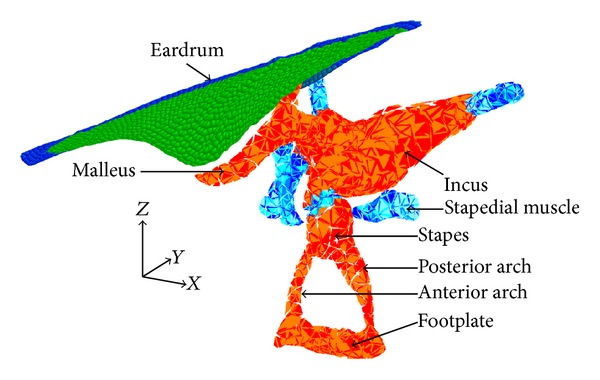
Finite element model of the middle ear structures. The tympanic membrane is composed of the pars flaccida, the pars tensa (green), and the tympanic annulus (blue). It is represented by triangles. Bones (red), ligaments, and tendons (blue) of the ossicular chain are modeled as tetrahedrons elements. Annular ligament is represented as springs and is attached to the footplate.

For the simulation, a FEM of the middle ear was developed and was computed at interactive rates (Figure 3). The mechanical behavior under physiological condition or under surgical stress assumptions was successfully confronted to human temporal bones observations [14]. To evaluate the level of realism of the model, we compared the results with two different measures. The first one was the evaluation of the transfer function of the ossicular chain, in the presence of an acoustic pressure wave, like in [12, 13, 15]. This test is similar to a clinical audiometry, which is used to evaluate the hearing thresholds or postoperative surgical outcomes. As the results of the transfer function of our FEM were in good accordance with the data measured on human temporal bones, it validated the dynamic behavior of the model at high frequencies. The second test consisted in the application of a high static pressure on the tympanic membrane in nominal and pathological cases, like in [16, 17]. This comparison with experimental data validated the deformation model in presence of large displacements as in a surgical situation. The analysis of the transfer function and the high static pressure applied to the tympanic membrane in nominal and in pathological cases was conducted to evaluate the mechanical realism of our approach. Additionally, we showed that the cochlea has a negligible effect on the mechanical behavior for frequencies below 250 Hz even when a large ossicular displacement is applied, such as situations encountered in surgical manipulation. Thereby, for surgical simulation, the cochlea could be removed, in order to reduce computation time without compromising the realistic behavior of the ossicular chain. A semiautomated algorithm allows the deformation of our model according to the anatomical dimension of the patient based on clinical imaging (cone beam computed tomography scan) [18].

The FEM was based on a geometric model obtained from a micromagnetic resonance imaging and developed for anatomical teaching [19]. The FEM was implemented in the simulation open framework architecture (SOFA, http://www.sofa-framework.org), an interactive simulation software dedicated to medical simulation [20]. A Phantom Omni device (Sensable, Wilmington, MA) with 6 degrees of freedom for positioning was used to interact with the simulation.

The ossicular chain was modeled by tetrahedral elements and the tympanic membrane by triangular (CST) elements. These constituted our mechanical atlas. Young's modulus and density parameters of all the components were set according to published data on human ear or by cross-calibration process and were reported in our previous publication [14]. The Rayleigh damping parameters were assumed to be *α* = 0 s^−1^ and *β* = 1.0 × 10^−4^ s and the Poisson's ratio was set at 0.3 for all middle ear components. The contact response between the instruments of the robot, the ossicular chain, and the prosthesis were computed using unilateral constraints and solved using a dedicated solver that could handle snap-in tasks [21]. In order to simplify the simulation, when grasping, the prosthesis was mechanically attached to the grasper of the robot with a generalized spring.

Computation efficiency is the key of a surgical simulator, as it needs to be interactive. Nevertheless, collision detection, constraints resolution, and mechanical deformation increase time computation. However, we did not choose to decrease the realism of the simulation. The goal of this project was to build a surgical simulator for teaching and rehearsal and to enhance our robotic device. As a consequence, our approach needed to be as realistic as possible. To achieve this goal, the validated FEM was used in combination with an implicit integration scheme and an asynchronous preconditioning technique, as presented in [22]. The preconditioner did not only improve the convergence of the conjugate gradient used to solve the FEM system, but also provided a way to speed up the computation of the contact constraints. All our simulations were performed on a conventional workstation, with a backward Euler scheme and a time step of 0.04 s. A 3D viewer HMZ-T2 (Sony, San Diego, CA) was used for 3D rendering of the virtual scene.

### 2.3. Surgical Procedures

One of the most challenging surgical procedures in the middle ear surgery is the deposit of the ossicular prosthesis as the surgeons are in direct contact with the ossicles. Indeed, an involuntary motion may results in severe damages to the structures and could yield irreversible total deafness. The simulated surgical step consisted in performing a stapedotomy followed by the placement of a prosthesis piston through the stapes footplate, called stapedioplasty. As the goal was to provide a rehearsal surgical simulation and a platform for the development and the evaluation of new tools and procedures, it was necessary to simulate realistically these critical steps.

#### 2.3.1. Stapedotomy

In conventional surgery, a stapedotomy consists in the perforation of the stapes footplate using either a laser or a surgical burr. The laser is a safe technic which does not need to be in contact with the ossicular chain. However this technic can raise the temperature of the footplate, which may damage the inner ear and postoperative hearing results. The drill does not have this drawback but the applied gesture needs higher accuracy. Indeed, if too much pressure is applied on the footplate, there is a risk to fracture the footplate or to push it into the inner ear (floating footplate), thus yielding complete deafness. For prosthesis with a 0.4 mm diameter, a 0.6 mm diameter hole is required. A too large opening in the footplate may induce a leak of the inner ear fluids postoperatively into the middle ear cavity, compromising the hearing.

The drilling operation was simulated using an algorithm derived from constructive solid geometry subtraction [23]. Therefore the operation required objects in which underlying geometries were based on signed distance fields. Given two of them, one called* Surface* and the other* Tool*, the computation below was done in order to update the distance field of the* Surface*:

(1) $$\begin{array}{c} Surface=\max\left(Surface,-Tool\right). \end{array}$$

**Figure 4:**
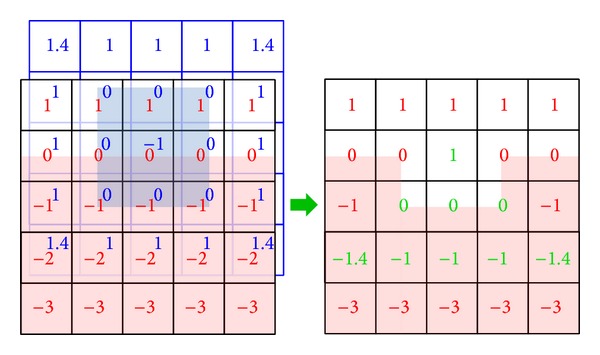
Application of the carving process with a cube as* Tool* (blue) and a plane as* Surface* (red) based on signed distance map. The outcome is displayed on the right side, and the local update is pictured in green.

The whole process was organized around one component whose purpose was to retrieve access to the distance fields and the corresponding collision models. The drilling process was activated by a peripheral input. So the collision between the* Tool* collision model and all the* Surfaces* was checked and the rightful surfaces according to the contacts are updated. More precisely, the update was made locally around the detected contacts to avoid unnecessary computation and to save time.  Figure 4 represents the updated values in green during the interaction of a* Surface* in red with a* Tool* in blue. The final rendering was currently carried out by the Marching Cubes algorithm, recreating meshes from the distance fields.

**Figure 5:**
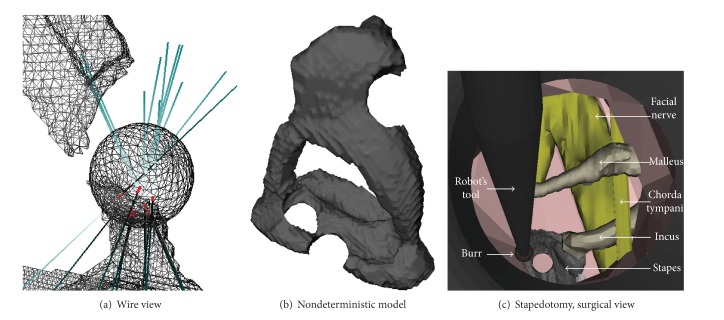
Simulation of a drilling procedure using a burr. (a) Contact interactions are represented in red and blue during a drill. (b) Represents the results of the marching cube algorithm. Any part of the bone can be drilled. (c) Surgical view of a stapedotomy simulation using the RobOtol.

This method was based on an unsettled model, which allowed the user to drill any part of the bones (Figures 5(a) and 5(b)). Thus an imprecise gesture or a misplacement of the stapedotomy by the resident may be simulated. Moreover to study and analyze new procedures or tools it was important to use a nondeterministic model for our simulation.

The collision detection required models that could compute the intersection between a distance field and different mesh primitives. The intersection with a point was based on the following idea: the position of the point inside the distance field can easily be computed and thus the presence of contact or not can be determined, including the distance to the surface of the object. The line and triangle intersections rested on the same basic concept, dividing the primitive in a set of points to compute the position of every one of them in the distance field. Other pairs of intersection have yet to be implemented.

To simulate the drilling surgical step, we used a sphere to represent a 0.6 mm diameter burr linked to a visual surgical hand piece. The temporal bone, the facial nerve, and a 6 mm diameter speculum were visually represented to reproduce surgical environment and visual obstacles (Figure 5(c)). Collisions were only computed between the burr and the ossicular chain. To complete the task, the surgeons had to drill the stapes footplate in order to obtain a 0.6 mm diameter hole. The goal of this task was also to evaluate the feasibility to drill the footplate of the stapes with a surgical hand piece mounted on the RobOtol.

The ideal position of the stapedotomy was determined initially by an expert surgeon and was not displayed to the user during the test. This optimal position was shown only at the beginning of the test during few seconds to avoid the divergence of the user position estimation. The goal was to determine user ability to reach a memorized target with the teleoperated system. The path of the robot, the distance of the burr to the optimal position, and the execution the time of the task were recorded.

#### 2.3.2. Placement of the Prosthesis

**Figure 6:**
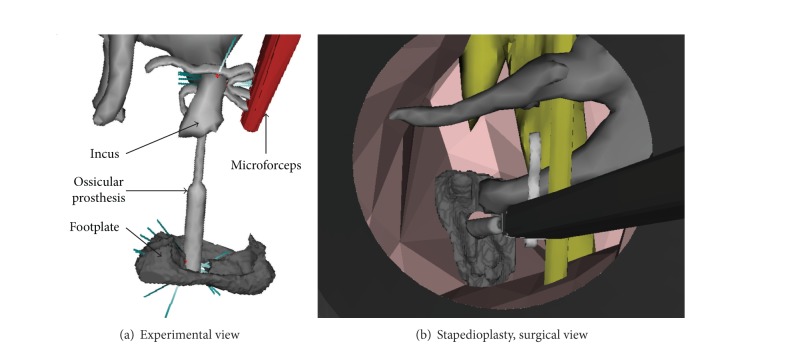
Simulation of the placement of an ossicular prosthesis. The ossicular prosthesis is placed through the perforated stapes footplate and fixed to the incus. (a) Experimental view with the visualization of the detected collisions in blue and red lines. The prosthesis is clipped to the incus. (b) According to a surgical view, the round gray area is the view of a 6 mm diameter speculum. The prosthesis is going to be clipped.

The crimping procedure of the prosthesis piston on the incus long process is an even more critical step. Involuntary movement on the ossicular chain may induce a rupture of the incus-malleus joint or incus luxation. Severe damage occurs when forces around 0.9 N in the anterior-posterior direction (*X*-axis in Figure 3) or 0.7 N in the lateral-medial direction (*Z*-axis in Figure 3) are applied [24]. A Soft-clip (Heinz Kurz GmbH, Dusslingen, Germany) prosthesis piston with diameter of 0.4 mm and length of 4.5 mm was modeled in the simulation as hexahedral deformable elements (Figure 6(a)). The density parameter and the Young modulus of pure titanium medical grade 2 prosthesis were set at 4.5 × 10^3^ kg/m^3^ and 344 MPa. A virtual prototype of a surgical microforceps designed for the RobOtol was implemented in Sofa.

The simulation of the stapedotomy was previously performed to drill a 0.6 mm diameter hole centered on the stapes footplate. The ablation of the stapes branches was performed by a total drilling of the superstructure of the stapes. This is not the conventional procedure since the separation of the stapes superstructure to the footplate is performed by bone fracture, but this step was not yet implemented in our simulator. Nevertheless, the resulting model was used with the same mechanical parameters for the simulation of the stapedioplasty surgery. The bottom of the ossicular prosthesis was first placed through the stapes footplate, and then the upper part was set upon the incus long process. Finally, the prosthesis was pushed until it was crimped on the incus (Figure 6(b)). This is the most critical surgical step because the required forces to fix the prosthesis are close to forces that can lead to incudomalleolar articulation rupture. The execution time and the incus displacement were recorded throughout the simulation of the procedure.

#### 2.3.3. Innovative Surgical Procedures

RobOtol enhances gestures accuracy and reduces physiological tremor. By taking advantage of these functionalities, new procedures could be performed using the robot. During the crimping of the prosthesis, the incus is moving due to the applied pressure required to push the prosthesis. To avoid this effect, an option consists in maintaining the incus using an instrument. This intervention, using two tools in contact with the ossicular chain at the same time, is almost impossible to perform in conventional surgery. The robot has the possibility to hold the same position of the tool with no tremor or tiredness effect compared to a manual surgery.

**Figure 7:**
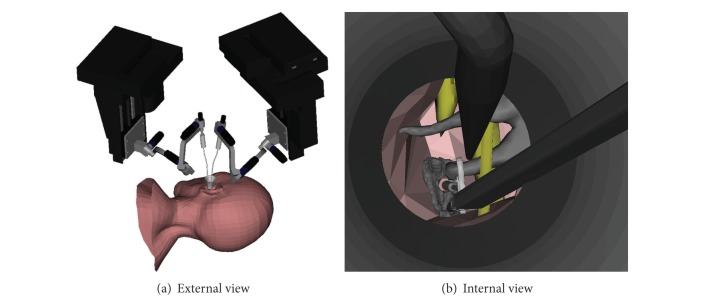
Middle ear surgical simulation using two robot arms simultaneously. (a) External view of the surgical scene. (b) Surgical view of a stapedioplasty procedure using two arms.

The idea was to use a second robotic arm equipped with a basic instrument, such as a microedge or a suction tool, and to place it close to the incus on the opposite side of the prosthesis approach. Thus during the crimping process the second robotic tool would restrain the incus motion inwards the middle ear cleft (Figure 7(b)). The hypothesis was to reduce the incus displacement to avoid damage of the incudomalleolar joint.

However, this procedure requires two robotic arms in contact with the ossicles through the 6 mm diameter speculum. To avoid collision between the arms and to preserve as much as possible the surgical visual field, the configuration of the two robot arms in the operating room was investigated. Again, the execution time and the incus motion were recorded during the experiment.

Results were expressed as mean ± standard deviation, an unpaired *t*-test was used to compare our results and were analyzed with *R* statistical software (http://www.r-project.org).

## 3. Results

Robot-based stapedotomy and ossiculoplasty were successfully conducted using a haptic device interface. More than 40 frames per second were observed with no interaction between tools and organs and have dropped to 20 frames per seconds when more than 30 collisions occurred. This was the limit of acceptance for real-time interactions, but we considered that it was sufficient enough for our application. The lowest number of frames rate observed throughout all our simulations is 10 Hz and is measured during strong interaction such as drilling with 80 simultaneous collisions. Motion and deformation of the ossicular chain during a palpation with the tools were subjectively reported to be realistic by the surgeons.

The drilling algorithm based on the signed distance fields coupled to a Marching Cube algorithm allowed performing a stapedotomy. Incomplete or abnormal drilling like elliptic shaped hole was possible. The mean execution time was 80 ± 17 s (*n* = 5) with a mean duration time of 39 ± 10 s related to the descent of the robot's tool in contact with the stapes footplate. A minimal distance of 0.07 ± 0.03 mm (*n* = 5) was observed between the target position of the stapedotomy and the different trials. The volume of the virtual stapedotomy was 0.11 ± 0.005 mm^3^ (*n* = 5), which corresponded to 7.3 ± 0.33% of the total volume of the stapes footplate that had been drilled.

Users were able to place the bottom of the ossicular prosthesis through the footplate with a microforceps and to place the upper part onto the incus inferior branch. The crimping process was assured by pushing the prosthesis like in manual surgery. The prototyped virtual tool preserved the surgical view (Figure 6(b)). Using a second robot's arm or a surgical hand piece mounted on the robot, surgeons were still able to move freely with the robot in the middle ear cavity. No obstruction was reported and the visual field of view was sufficient to practice surgery. No collisions between the arms, located at ±120 degrees compared to the surgeon's position, were detected (Figure 7(a)).

The mean duration time of the total stapedioplasty was 140 ± 54 s (*n* = 5) for the experiment with a single tool and 175 ± 40 s for the task with the second robot arm. The movement of the incus was 0.24 ± 0.09 mm (*n* = 5) when using a single microforceps and 0.10 ± 0.05 mm (*n* = 5) when using two tools simultaneously. A decrease displacement of the long branch of the incus was observed, while a second tool was placed against the incus (*P* = 0.025, student). A sharp decrease of the motion along the *X*-axis (Figure 3) was observed (0.15 ± 0.05 to 0.05 ± 0.02 mm, *P* = 0.008, student) as well as along the *Y*-axis (from 0.12 ± 0.04 mm to 0.06 ± 0.03, *P* = 0.029, student). However, the use of a second tool did not reduce the incus displacement along the *Z*-axis (0.16 ± 0.12 mm for the method with a single tool against 0.07 ± 0.06, *P* = 0.198, student).

## 4. Discussion

The interactive simulation presented in this paper was developed for training, rehearsal, and improving the new robotic procedures. The simulation was performed using a physics-based validated model, which ensured the realism of the mechanical behavior of the middle ear [14]. In addition to the physiological and surgical condition evaluation, the behavior of our FEM was described as realistic by expert surgeon during a palpation of the middle ear structures with the simulated robotic tools. The optimization process, implemented in SOFA, allowed simulating simultaneous interactions between the ossicular chain, a deformable prosthesis and two robot's arms controlled by an operator, at an average frame rate of 20 frames per second. However, interactions involving a relatively large number of contact points reduced the computational efficiency, suggesting that further optimizations will be needed in the future.

Two challenging procedures of the middle ear surgery were successfully performed using the RobOtol with our simulation approach: stapedotomy and stapedioplasty surgery. An unsuccessful procedure, such as misplacement of the burr during a stapedotomy, could be simulated and evaluated by the analysis of the shape of the hole or the applied forces on the footplate. For instance, as the drilled volume of the stapes footplate was relatively reproducible, an abnormal volume can indicate a bad performance. On the contrary, good achievement of those tasks validated the cinematic efficiency and abilities of the robot to perform middle ear surgery. Moreover, our simulation could constitute an alternative to temporal bone dissection for surgical training. Indeed, abundant training session is a real issue because of the diminishing availability of human temporal bones [25, 26].

The execution time of those two robot-based procedures was longer than in conventional surgery but within expected values when performing such difficult procedures. It should be noted that the duration time of the stapedotomy was rather repeatable, although it was not the case for the stapedioplasty. The high level of difficulty to perform those procedures can explain this observation. These approaches require a good positioning adjustment to place the prosthesis, which can take a variable amount of time. When using two arms at the same time, the execution time was longer. This delay of 35 s was due to the placement of the second robot's arm, although it is possible to move the two robot's arm simultaneously with two master arms. Nevertheless, the additional duration of the task was not excessive compared to the mean duration of a complete otosclerosis surgery, which is reported as 54 ± 21 minutes [27].

Between the different stapes drilling simulations, the target position was reached with 0.07 ± 0.03 mm accuracy on a footplate of 2.8 × 1.52 mm (elliptic shape) with a 0.6 mm diameter burr. This error relies on the memory capability of the surgeon as well as the accuracy of the teleoperated system. It means that the surgeons were able to command the robot's tool to a desired position with a mean accuracy of 0.07 mm. The drilled volume observed was around 7.3% of the total volume of the stapes footplate with a standard deviation of 0.33. That means that our experiment was very repeatable and that the robot is accurate enough to conduct a stapedotomy.

The size of the surgical burr was very important compared to a micropeak. When mounted on the robot's arm, its visual obstruction was added to the robot itself. It was important to investigate the tool arrangement in order to preserve the visual field. The different simulations suggest that the visual obstruction of the surgical burr was not a major issue, since no discomfort was reported by surgeons using the simulator.

The placement of the prosthesis using the robot was successfully simulated, with either a single or two instruments. The results suggested that we were able to reduce the global displacement of the incus during the surgical procedure by 57% with a second arm to maintain the long process of the incus. Figure 7(b) represents the surgical view using two simultaneous arms. In conventional surgery, this procedure is almost impossible to accomplish, as the manipulation of two tools simultaneously in contact with the ossicular chain requires perfect hands coordination and dexterity. The incus motions on the anterior-posterior and on the upward-backward direction were reduced by 65%. The motion on the *Z*-axis (Figure 3) was not significantly changed with the new approach. This could be explained by the fact that the second tool did not constrain the incus in that direction. Therefore, a new tool, similar to a microhook, could be used to decrease the displacement of the incus in that specific direction. This example of a procedure comparison, evaluated using the simulation, shows that potential risk of incus luxation or fracture during piston crimping could be reduced using a second tool simultaneously. Obviously, this could be easily performed with two robotic arms.

The simulation and the results suggested that it was possible to improve the procedures using a robot-based system. Indeed a robot-based procedure could improve accuracy, ergonomics, fatigue, and tremor leading to increased safety of the patient and surgical outcome. Thus, our results demonstrated that two robotic arms at the same time could be used in order to decrease the risk of incus luxation or fracture. To do so, we have used the interactive simulation to test and evaluate a new surgical procedure using RobOtol.

One major middle ear surgery constraint is the bleeding phenomena. The blood fills the middle ear cleft from the bottom leading to an obstruction of the surgical workspace. The surgeons have to suck the blood using a suction cannula with their hands. However, it is very complicated to operate simultaneously with two tools through the external ear meatus. Several gestures, such as manipulation of the ossicular chain, may require both hands to stabilize the effective tool to control tremor. A second robotic arm could be placed at the bottom of the middle ear cavity for suction purpose. We showed that protection of the incus could reduce the ossicular chain motion. Thereby, to maintain the position of the incus while crimping the prosthesis, we can use a nonspecific shaped tool. The dimensions of this tool are compatible with usual suction cannula used in middle ear surgery. Thus, as a future work, we will study the possibility to use one tool mounted on a second arm to reduce bleeding embarrassment and to maintain the incus during real manipulation of the ossicles.

The simulation could also be used to train the physicians to the use of the robot and to evaluate the quality of the simulated procedure based on four evaluation criteria. The first criterion is based on the anatomical point of view, with the study of the shape, or placement of the hole in the footplate or the prosthesis on the incus compared to expert intervention. The analysis of the applied forces on the anatomical structures could be the second criterion, which allows the assessment of the surgical gesture ability. The visual obstruction duration could be taken into account to evaluate the procedure, as a third criterion. And finally the quality of the functional results of the surgical procedures could be estimated by the differential analysis of the ossicular transfer function results before and after the intervention as a fourth criterion. Equation (2) illustrates our assessment process that could provide an objective evaluation of a performed procedure. Thereby, the robot's tool prototype and different surgical approaches can be compared:

(2) $$\begin{array}{c} E=\alpha \frac{1}{S}+\beta \frac{1}{F}+\gamma \frac{1}{\text{VF}}+\delta \frac{\text{T}{\text{F}}_{\text{after}}}{\text{T}{\text{F}}_{\text{before}}}, \end{array}$$

where *α*, *β*, *γ*, and *δ* are constants; *S* expresses the distance to an optimal position initially determined by an expert surgeon; *F* takes into account the force applied on the anatomical structures such as in (3); VF corresponds to the visual obstruction time; and TF represents the transfer function results with the detailed computation reported in [14] and corresponding to the surgical outcome estimation. The execution time is not represented because we believe that it has no negative effect on the surgery result:

(3) $$\begin{array}{c} F=\mu \frac{df}{dt}+\lambda \int f\;dt. \end{array}$$

Other contribution of this work is to offer the possibility to evaluate new tools design for our robotic system. Interactive simulation allows the surgeons to determine whenever the tool is obstructing the field of vision during the surgical intervention and to evaluate their efficiency. Based on this work, questions about the instruments are raised, such as the better side to open the microforceps jaws. Our simulation offers the possibility to see and feel what could be the surgery using these tools, thus assessing the difficulty to perform it. Thus, the development cost and time could be reduced and direct feedback from the surgeons could be taken into account during the design process.

To perform a stapedioplasty with the RobOtol, a new actuator, providing 7th degree of freedom, should be implemented in order to grasp the prosthesis. The number of mobile jaws is now set to one in order to avoid damage on anatomical structures while opening the forceps. This new degree of freedom is compatible with the current RobOtol design and can be ensured by a step motor AM 1020-A0.258 (Faulhaber GmbH, Germany). Buttons on the master arm device or on the pedal-board can control opening or closing action of the forceps. A real version of the forceps for the robot will be built and based upon the tool evaluated with our interactive simulator. Furthermore, we know that the robot's actuators are powerful enough to handle a surgical hand piece without any change of the motion performance of the robot. However, before mounting a surgical hand piece on the robot's end effector, we have to verify that the burr vibrations will not disturb the robot and its accuracy. This will now constitute our next work. The interactive simulations presented in this work allowed performing a preliminary test on the feasibility and on the design of robot-based procedures using the robot.

At any time, the robot was able to complete the procedures and did not require a manual intervention. As the robot has no force sensor, no haptic rendering was transmitted to the user. The surgeon estimated the proximity and the contacts between the virtual components with the 3D rendering using the 3D viewer and visualization of the anatomical structures displacement or deformation like in actual surgery. Results showed that the robot was compatible with these surgical procedures, even without force feedback as in the current configuration of the robot, and that the physicians can be trained to the use of the robot by the simulation.

## 5. Conclusion

The clinical tests of our teleoperated system, called RobOtol, will start in few months. To accustom the surgeons to the robot and to investigate new tools design and new surgical procedures, we developed a surgical simulator of the middle ear microsurgery. Unlike most training system, the purpose here was to use the simulation for rehearsal procedures that will later be performed using a robotic system, called RobOtol. Thereby, we showed that our simulator allowed the design improvement of this teleoperated system such as the addition of new degrees of freedom and also prepared the physician to the first clinical interventions that will be performed with the robot. To reach this goal, the simulation was based on a validated finite element model of the middle ear, compatible with real-time computation. Moreover, interactive drilling was implemented and based on a nondeterministic model. Two challenging middle ear surgeries, a stapedotomy and a stapedioplasty, were simulated using the RobOtol. To our knowledge, virtual simulations of those microsurgical procedures had never been reported yet.

Moreover, the concept of using a real-time simulator to test and evaluate the new possibilities given by the robot and to design the robot tools was also original. Currently, we have already investigated the ability to use easily two robotic arms simultaneously during the robot-based procedure.

Further surgical simulations using the RobOtol will allow the development of a set of tools and to test innovative surgical procedures in order to improve surgical outcomes and patient safety.
